# Supplementary material for: Strain to shine: stretching-induced three-dimensional symmetries in nanoparticle-assembled photonic crystals
Source: Nat Commun. 2024 Jun 18;15:5215. doi: 10.1038/s41467-024-49535-z (PMC11189559; doi:10.1038/s41467-024-49535-z)
Supplement: Supplementary file 1 — Supplementary Information [file 41467_2024_49535_MOESM1_ESM.pdf]

Supplementary Information for

Strain to shine: stretching-induced three-dimensional symmetries in nanoparticle-assembled photonic crystals

Tong An[1], Xinyu Jiang[1], Feng Gao[1], Christian Schäfer[2], Junjun Qiu[1], Nan Shi[1], Xiaokun Song[1], Manyao Zhang[1], Chris E. Finlayson[3], Xuezhi Zheng[4], Xiuhong Li[5], Feng Tian[5], Bin Zhu[6], Tan Sui[6], Xianhong Han[7], Jeremy J. Baumberg[8*], Tongxiang Fan[1*], Qibin Zhao[1*]

1. State Key Lab of Metal Matrix Composites, School of Materials Science and Engineering, Shanghai Jiao Tong University, Shanghai, 200240, China
2. BASF SE, Dispersions & Resins, Carl-Bosch-Strasse 38, Ludwigshafen/Rhein, 67056, Germany
3. Department of Physics, Prifysgol Aberystwyth University, Wales SY23 3BZ, UK
4. Department of Electrical Engineering, KU Leuven, Leuven, B3001, Belgium
5. Shanghai Synchrotron Radiation Facility, Shanghai, 201204, China
6. School of Mechanical Engineering Sciences, University of Surrey, Guildford, GU2 7XH, UK
7. Institute of Forming Technology and Equipment, School of Materials Science and Engineering, Shanghai Jiao Tong University, Shanghai, 200240, China
8. Department of Physics, University of Cambridge, JJ Thomson Ave, Cambridge, CB3 0HE, UK

*Qibin Zhao

Email: zhaoqibin@sjtu.edu.cn

*Tongxiang Fan

Email: txfan@sjtu.edu.cn

*Jeremy J. Baumberg

Email: jjb12@cam.ac.uk

This PDF file includes:

Supplementary Figures S1 to S25

Supplementary Discussions 1-2

Movie S1-S3

Supplementary Reference

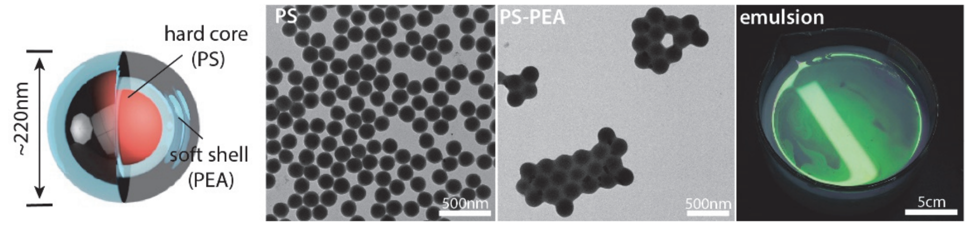

Figure S1. Core-shell sphere synthesis. From left to right: schematic representation of the PS-PEA core-shell sphere, TEM image of the PS core particle, TEM image post-PEA shell grafting, and the resultant core-shell sphere emulsion exhibiting the 'milk skin' effect colours indicative of good size monodispersity.

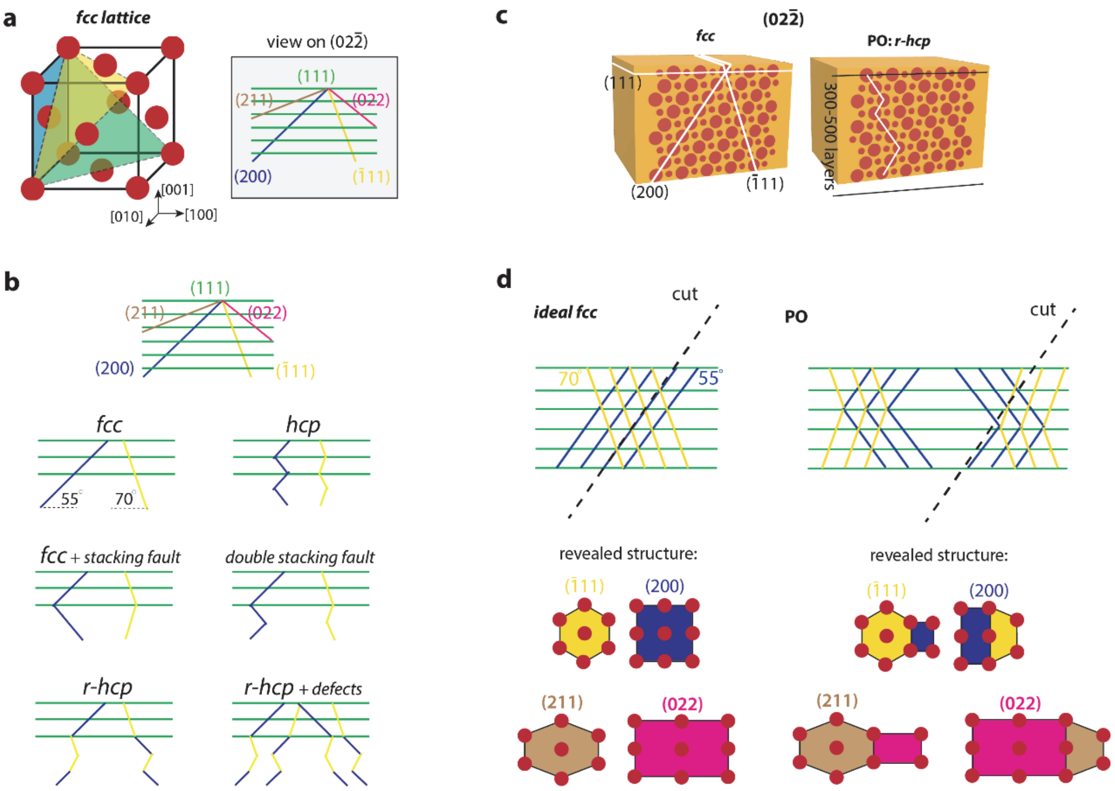

Figure S2. Contrast between different types of close-packing lattices. a, The cubic unit cell and the planes of the *fcc* lattice. **b**, Twisting of the plane orientations by stacking faults in different lattices. **c**, Illustrations of the orientations of (111), ($\bar{1}$11) and (200) planes in a *fcc* lattice and the *r*-hcp lattice of POs. **d**, In-plane packing structure of different lattice planes in *fcc* and *r*-hcp.

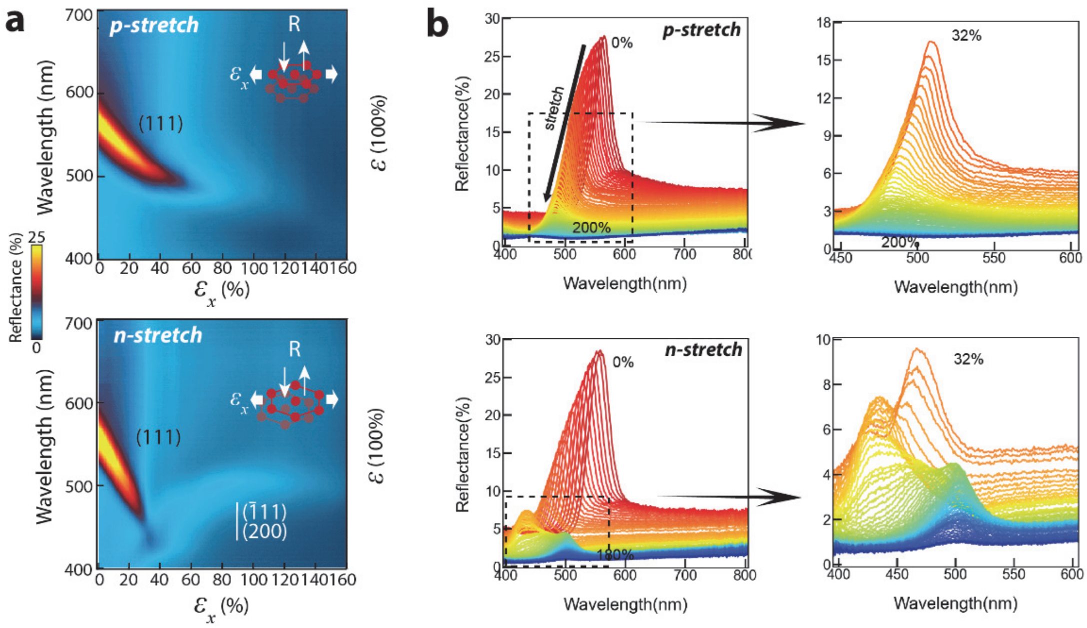

Figure S3. **Brightfield reflectance spectra of *p*- and *n*-stretched POs at normal incidence**. **a**, Evolution of the reflection spectra with strain. **b**, Raw spectra of **a**.

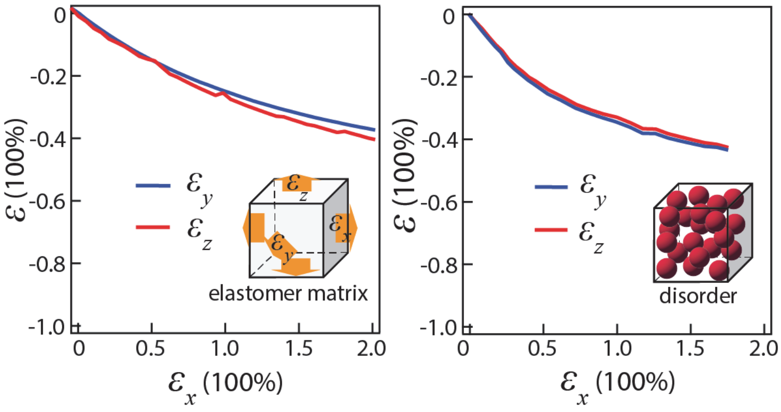

Figure S4. Thickness and width strains measured in poly (butyl acylate) (PBA) (left) and disordered PO (right). PBA was employed to bypass the pungent smell of PEA. Although PBA is softer than PEA, both are anticipated to show similar deformation characteristics.

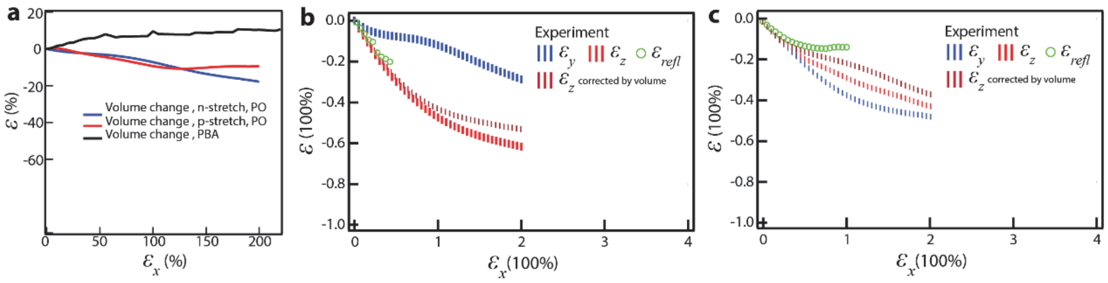

Figure S5. **Analysis of experimental errors in strain measurements**. **a**, Computed volume alterations in POs and PBA during *n*- and *p*-stretching using the measured values of ε_x, ε_y and ε_z. **b**, Adjustment of thickness strain based on volume fluctuations in *n*-stretching. **c**, Adjustment of thickness strain

considering volume fluctuations during p-stretching. It's pertinent to note that minor deviations in microscopic strain evaluations are inherent, especially given the extensive stretching strains employed. While the total material volume may exhibit slight variations during stretching due to numerous factors[1,2], for this analysis, we consider the most stringent scenario where these volume changes are entirely attributed to experimental inaccuracies. In practice, errors might be present in both width strain ε_y and thickness strain ε_z, with ε_z typically posing more challenges in accurate measurement. In a conservative assessment, we assume all discrepancies lie in ε_z, and subsequently adjust the measured ε_z using the calculated volume changes. The results remain consistent with our primary conclusions.

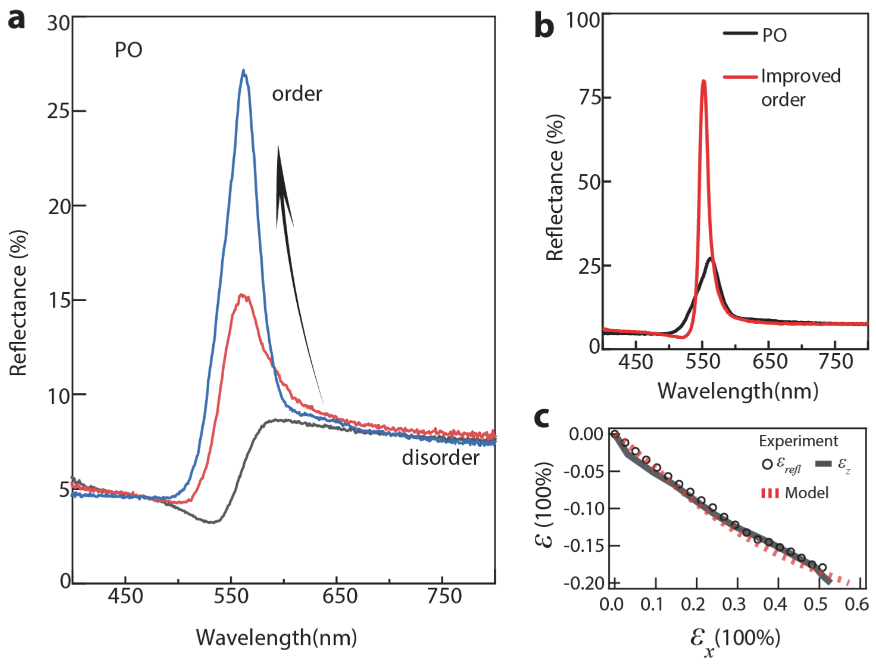

Figure S6. **Reducing the deviation between optical shift and thickness strain with improved structure order**. **a**, Reflectance spectra of POs under normal incidence with increasing structural order, captured using a 5X objective in brightfield mode; a noticeable blueshift emerges as structural order improves. **b**, Reflectance spectra comparison between PO and an elastic opal that we developed recently with much better structure order. **c**, Optical strain shifts ε_{refl} and thickness strains ε_z for the newly developed elastic opal in n-stretching; results align remarkably with model prediction thickness strain.

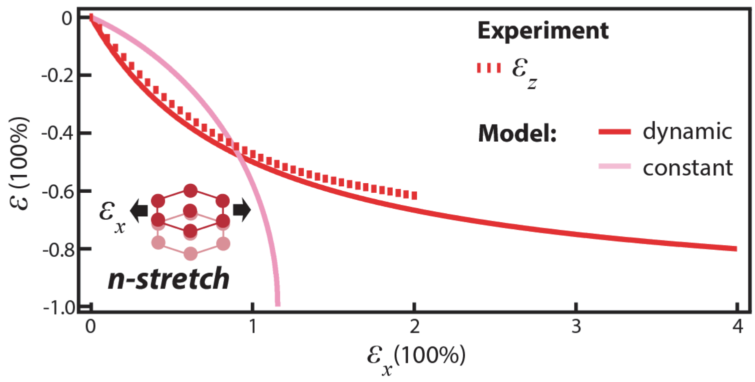

Figure S7. Measured and model predicted thickness strains in *n*-stretching. Thickness strains calculated from our model using a dynamic interparticle distance and the that of plasticity and solid-solid transitions using a contact interparticle distance are compared with the experimental result.

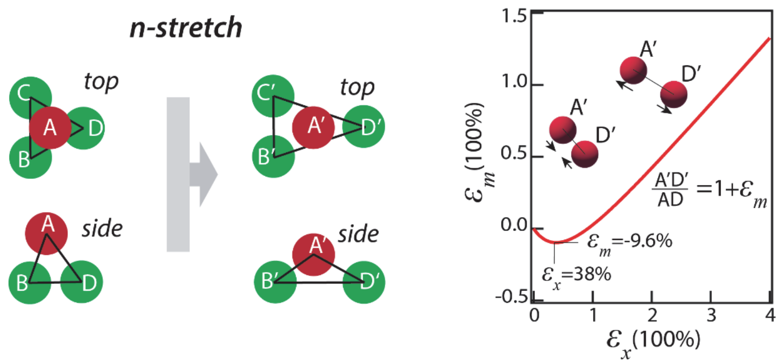

Figure S8. Distance changes $\varepsilon_m$between the apex sphere A and bases spheres represented by sphere D in *n*-stretching.

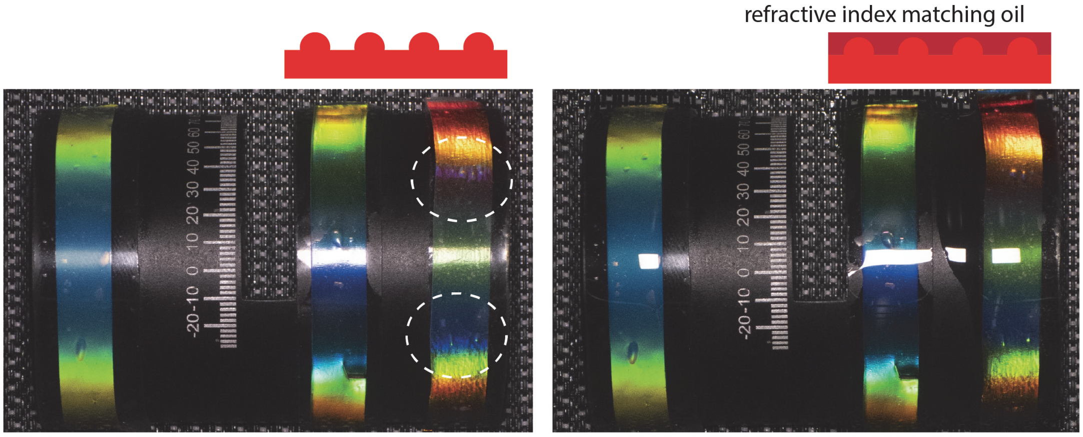

Figure S9. Retro-reflection colours of different *n*-stretched samples before and after immersing the surface with dimethicone oil. Three different samples are used, the sample on the left is 80% *n*-stretched PO, middle is the 80% *n*-stretched new green elastic opal, the new green elastic opal gives a reflection peak at ~550nm at normal incidence before stretching, very close to that of the PO, the sample on the right is a 80% *n*-stretched new red elastic opal, its reflection peak wavelength at normal

incidence before stretching is ~610nm. The sample are attached to a cylinder to we can view their retro-reflection colours from the top. The top surfaces of the samples are exposed to air. After coating the surface with a thin layer of dimethicone oil, which has a refractive index of ~1.4, surface diffraction colour indicated as the blue hue is significantly weakened. All the sample still show strong retro-reflection colours with oil on the surface.

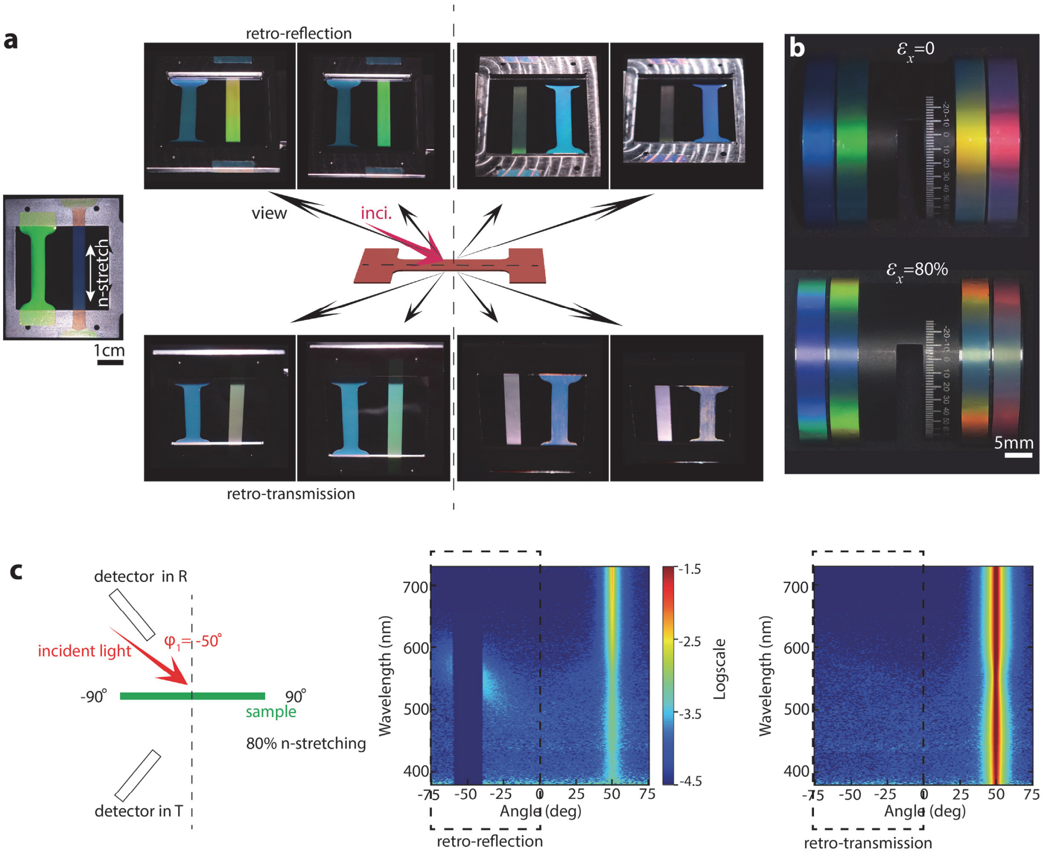

Figure S10. **PO's colour variations in different viewing angles pre and post *n*-stretching**. **a**, Left, colours of the unstretched sample and a sample post 80% *n*-stretching at normal incidence, panels on the right reveal different colours of the samples when observed at varied angles under oblique white light LED illumination. The retro-transmission colours are mainly due to diffraction of the incident light by (111) gratings in transmission, they show different brightness compared to the retro-reflection colours. **b**, POs of different particle sizes before and after *n*-stretching, samples are attached on to a cylinder surface. **c**, Contrast between measured scattering spectra in retro-reflection and retro-transmission for 80% strain *n*-stretched PO at a fixed incident angle of -50˚.

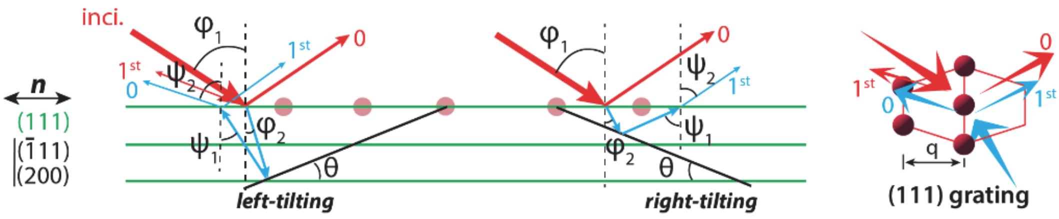

Figure S11. BCD model's reflection mechanism. Tilted planes are delineated by black lines. Blue represents the Bragg reflection from titled planes, while red signifies diffraction from the incident light by (111) gratings. (111) are volume gratings, but only surface-located spheres are depicted to avoid being cluttered.

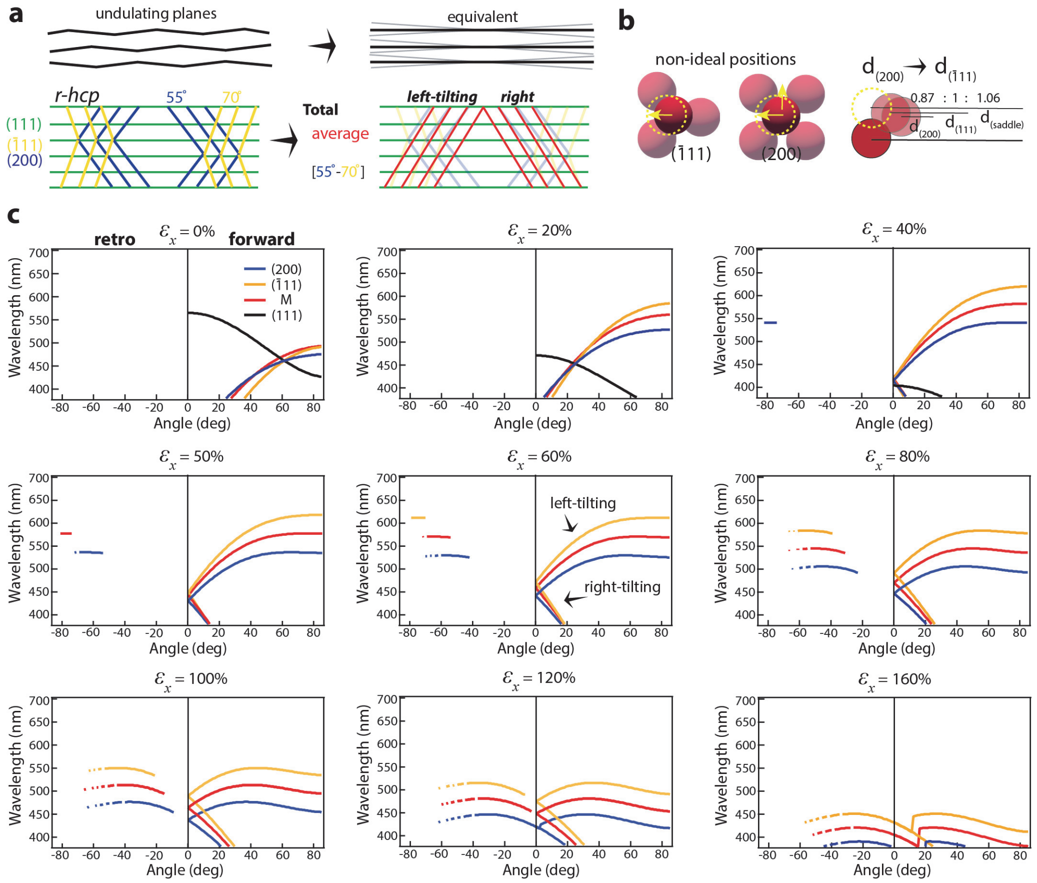

Figure S12. **BCD model predictions on Bragg reflection spectra of different lattice planes in the forward and retro-reflection directions**. **a**, Construction of the 'M' plane using the average plane distance and tilting angle of $(\bar{1}11)$ and (200). **b**, Illustration showing how spheres, when minutely shifted from their optimal positions within an imperfect lattice, lead to proximate plane distances for $(\bar{1}11)$ and (200) planes. **c**, Calculated Bragg reflections of different lattice planes at varied incident angles for *n*-stretched PO, 2D diffraction from the surface structure is not shown in the calculation because at each strain its spectrum varies according to the incident angle.

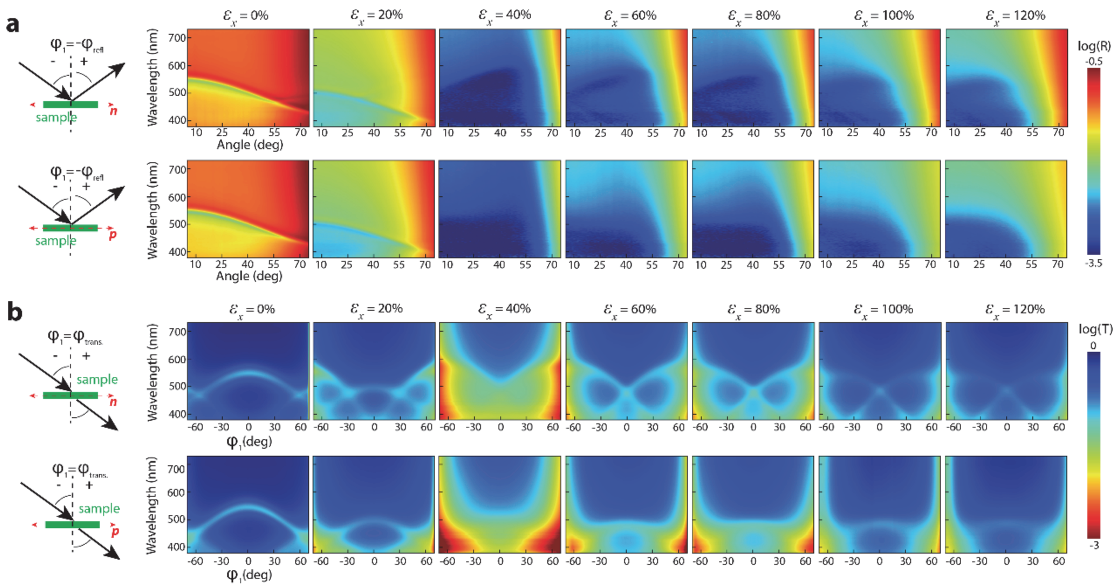

Figure S13. **Specular gonio-reflection and angular transmission spectra of *n*-stretched POs. a**, Specular reflection with incident light along n and p directions of *n*-stretched samples. **b**, Transmission spectra with incident light along n and p direction of *n*-stretched samples.

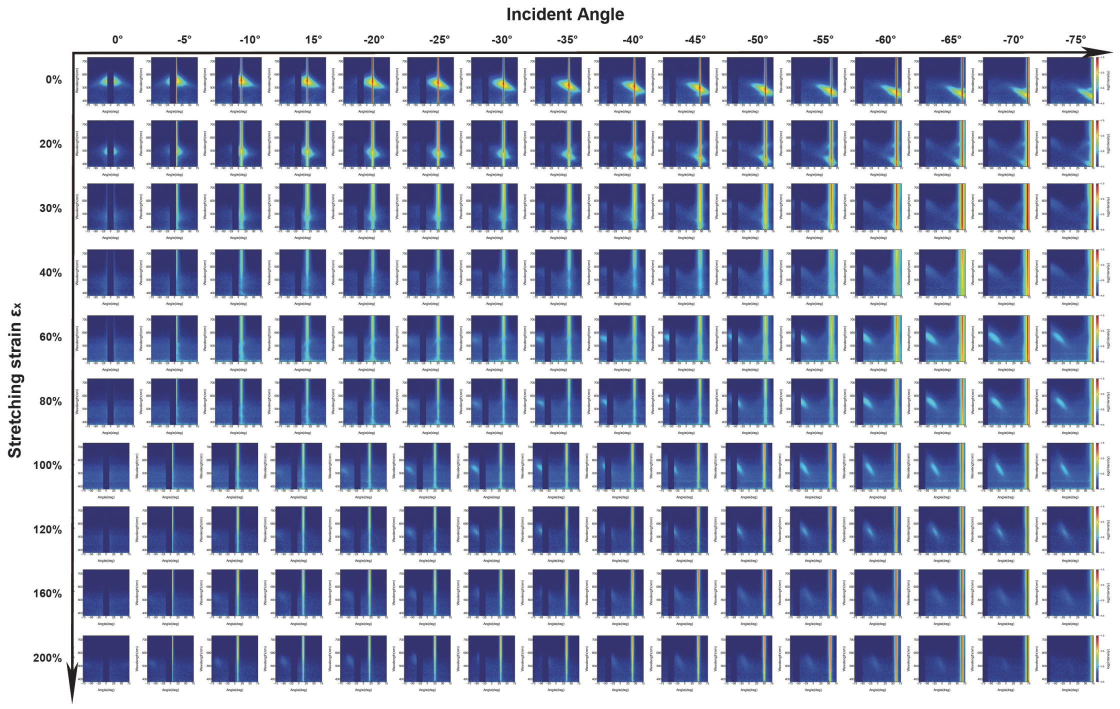

Figure S14. **Measured scattering spectra of *n*-stretched PO samples at varied incident angles along *n*-direction in both reflection**. Measured scattering spectra in reflection showing a more complete map of optical property transition with obvious retro-Bragg reflection spots.

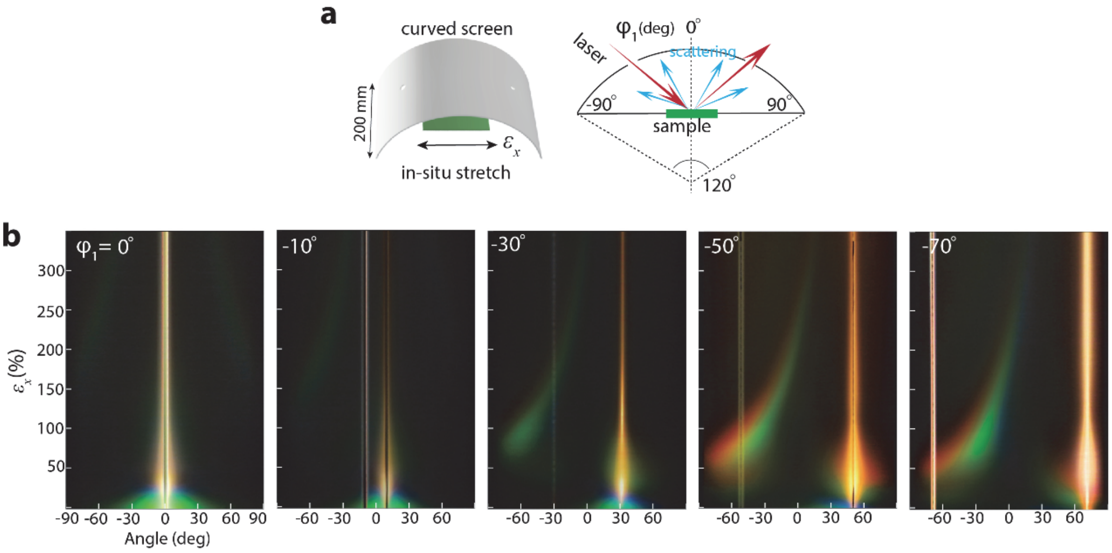

Figure S15. Angular scattering colour imaging of continuously *n*-stretched samples captured at different incident angles. **a**, Experimental conditions. **b**, Incident light is a supercontinuum white light laser, colours are true captured colours by imaging the screen.

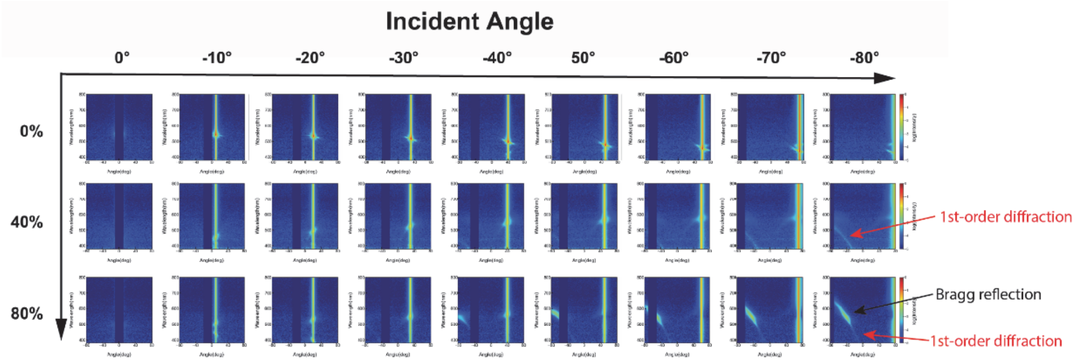

Figure S16. **Angular scattering spectra of *n*-stretched elastic opals with improved structure order**. The spectra reveal a precise overlap between the 1st-order diffraction of the incident light and the retro-Bragg reflection emanating from tilted planes. The samples are fabricated with regular size spheres, which produces ~550nm reflection peak at normal incidence similar to that of POs used in this work.

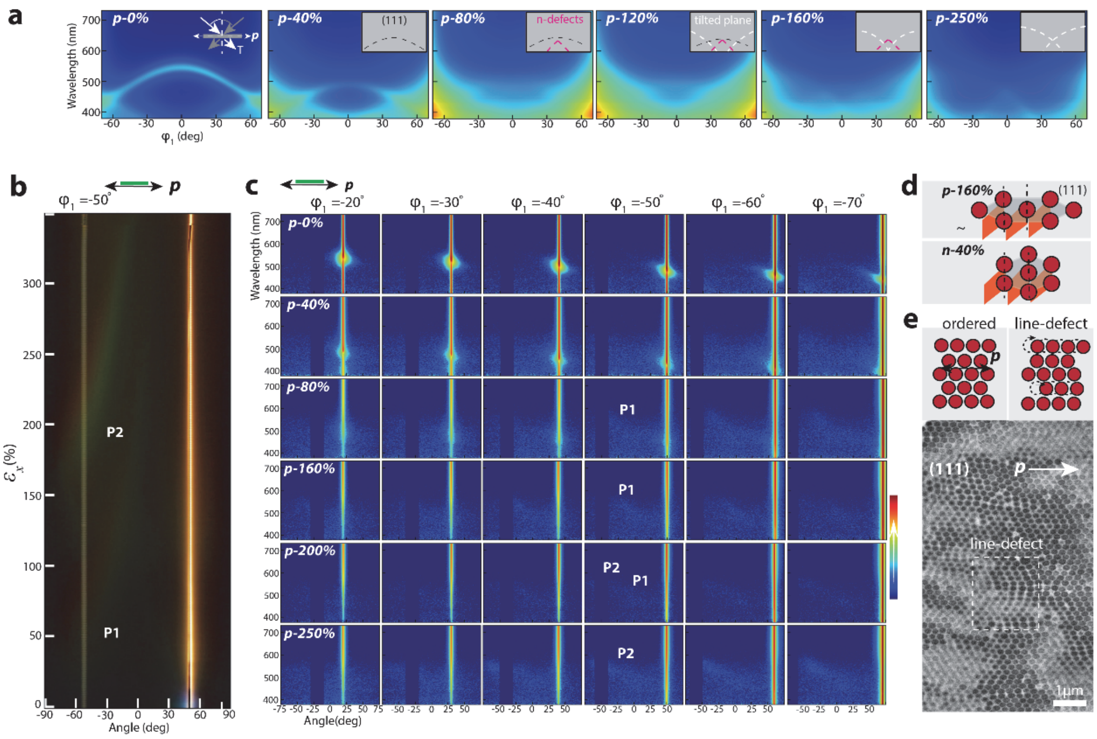

Figure S17. **Measured transition in angular optical properties of *p*-stretched POs**. **a**, Angular transmission spectra with light incidence along the *p*-direction. Insets detail the hypothesized structural origins of spectral features: black signifies the (111) planes, red highlights features from defects in (111) oriented in the *n*-direction, and white marks features from tilting planes. **b**, Colours scattered at varied angles during *p*-stretching at a -50˚ incident angle along the *p*-direction. The P1 colour band emerges from defects within the (111) planes oriented in the *n*-direction, while P2 represents colours from tilted planes. **c**, Scattering spectra from *p*-stretched samples under changing incident angles along the *p*-direction. Signals correlating with the P1 and P2 colour bands are highlighted. **d**, An illustration indicating the tilt angle of planes post 160% *p*-stretching, which closely aligns with that of a sample stretched in the *n*-direction at 40% strain. e, Evidence illustrating the presence of defects oriented in the *n*-direction within the (111) planes, stemming from sphere misalignments.

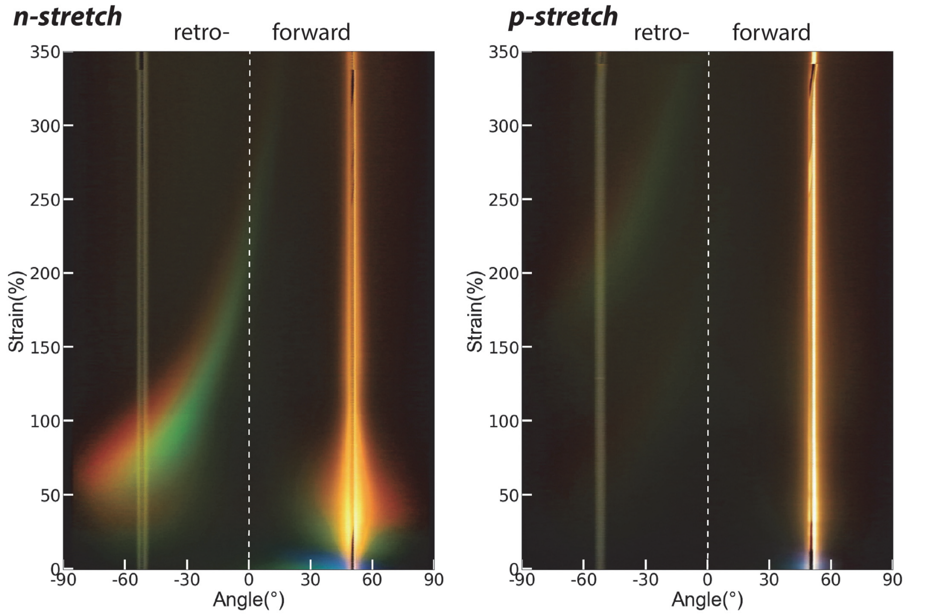

Figure S18. Contrast of the colours scattered at varied angles during *n*- and *p*-stretching at a -50° incident angle.

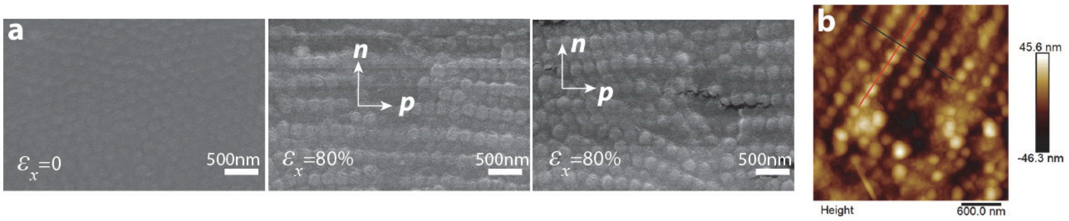

Figure S19. **SEM and AFM images contrasting the surfaces of unstretched and *n*-stretched POs**. **a**, SEM images of the surface structure deformation. Before stretching, the PS spheres are largely encapsulated in PEA, yielding a smooth surface. **b**, AFM depiction of a surface after 80% *n*-stretching in POs. This shows a sphere 'pop-up' phenomenon, with an average elevation of roughly 40nm.

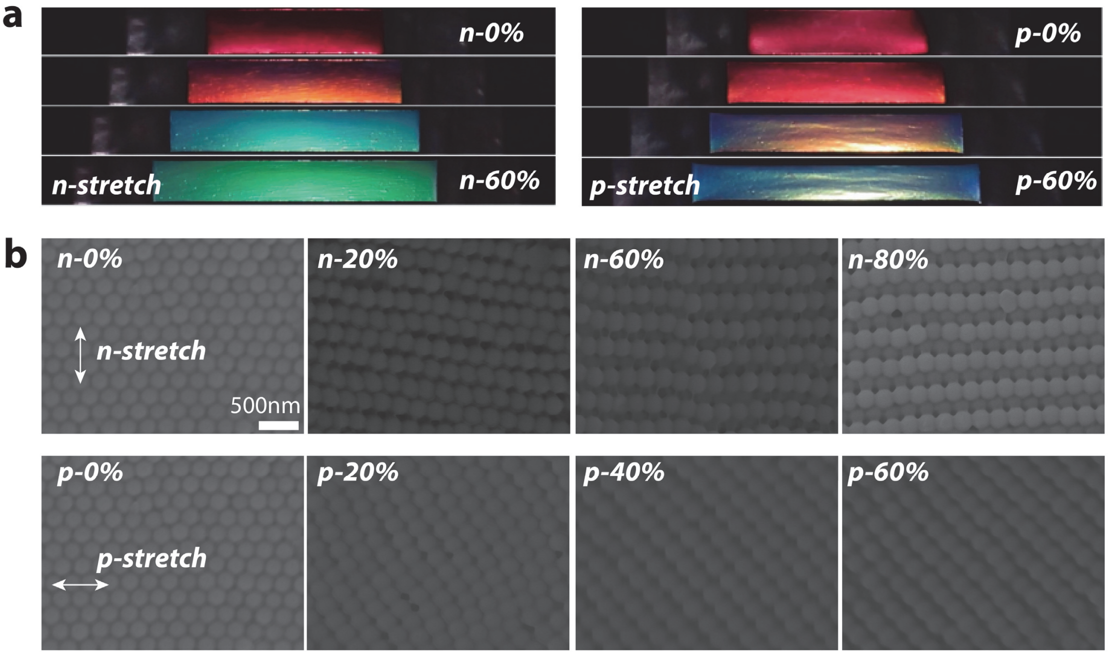

Figure S20. Stretching-induced changes in colours at normal incidence and the arrangements of spheres at the surface using new elastic opals of improved structure order. **a**, Left, colour change in *n*- and *p*-stretching, the original Bragg reflection peak at normal incidence is at ~650nm. **b**, SEM images of the surface structures in *n*- and *p*-stretching at different strains.

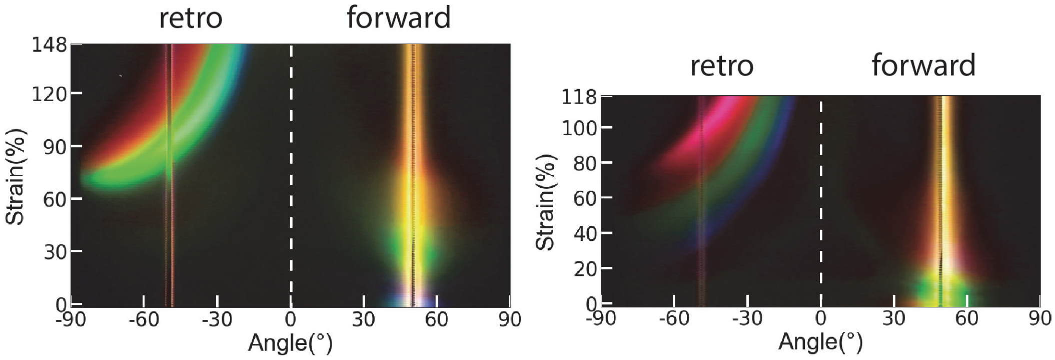

Figure S21. Angular scattering colour of a continuously *n*-stretched new elastic opals. **Left**, scattering colours of a new green elastic opal, the peak reflection wavelength at normal incidence is ~550nm, which is similar to that of the green POs used in our work. **Right**, scattering colours of a new red elastic opal, the peak reflection wavelength at normal incidence is ~650nm. The images were captured at φ_1= -50° incident angle, incident light is a supercontinuum white light laser, colours are true colours by imaging the screen

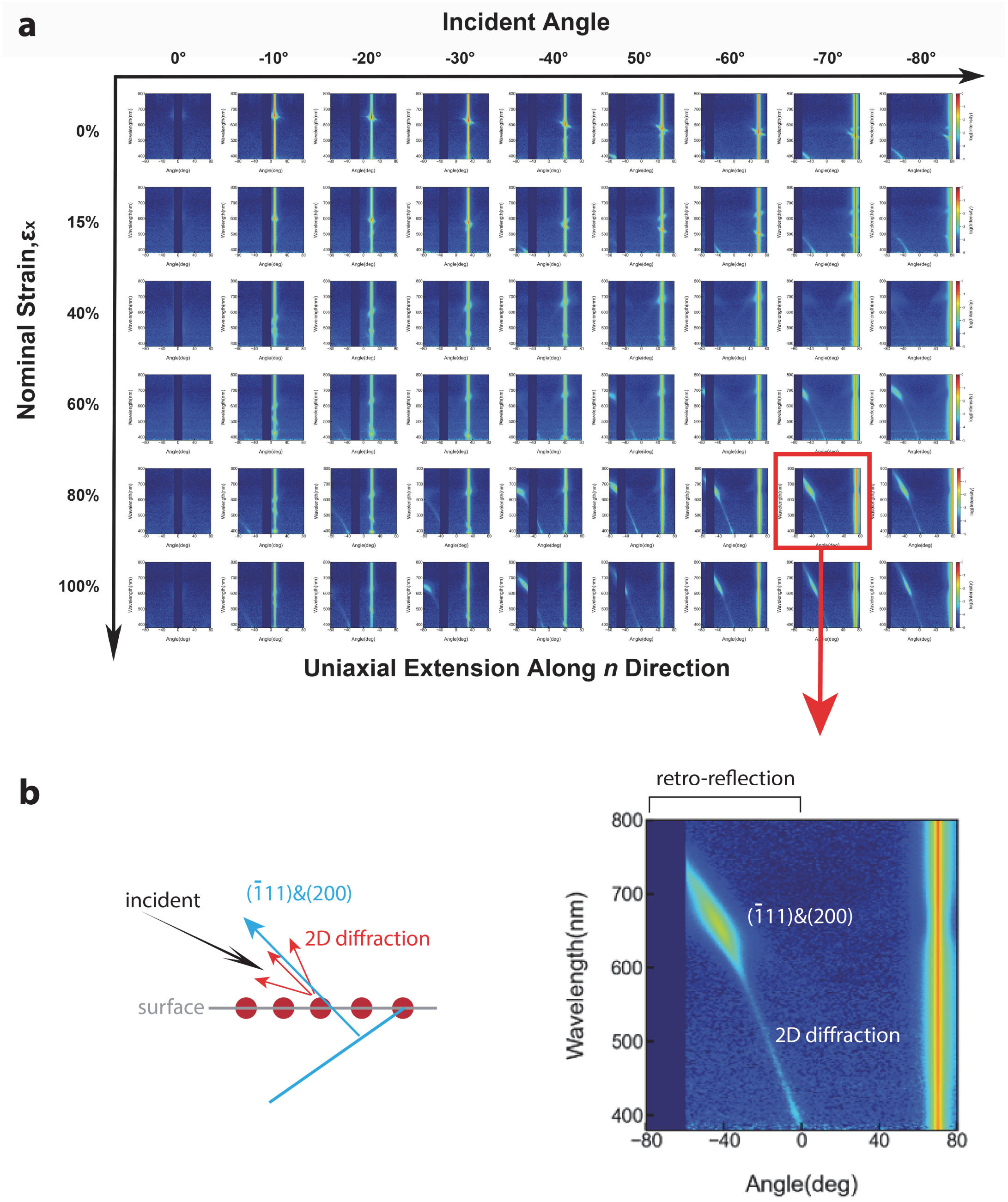

Figure S22. Angular scattering spectra in reflection for *n*-stretched new red elastic opal varying in incidence angle and strain. **a**, Measured scattering spectra at different angles in reflection at varying incidence angle and strain. **b**, The zoom-in of the angular scattering spectra at -70˚ incidence angle for 80% strain *n*-stretched sample. The optical mechanism is shown on the left, incident light is indicated as the black arrow, diffraction from the surface grating is indicated in red, the retro-Bragg reflection from the tilted planes is indicated in blue. The original Bragg reflection wavelength of the red elastic opal at normal incidence is at ~650nm.

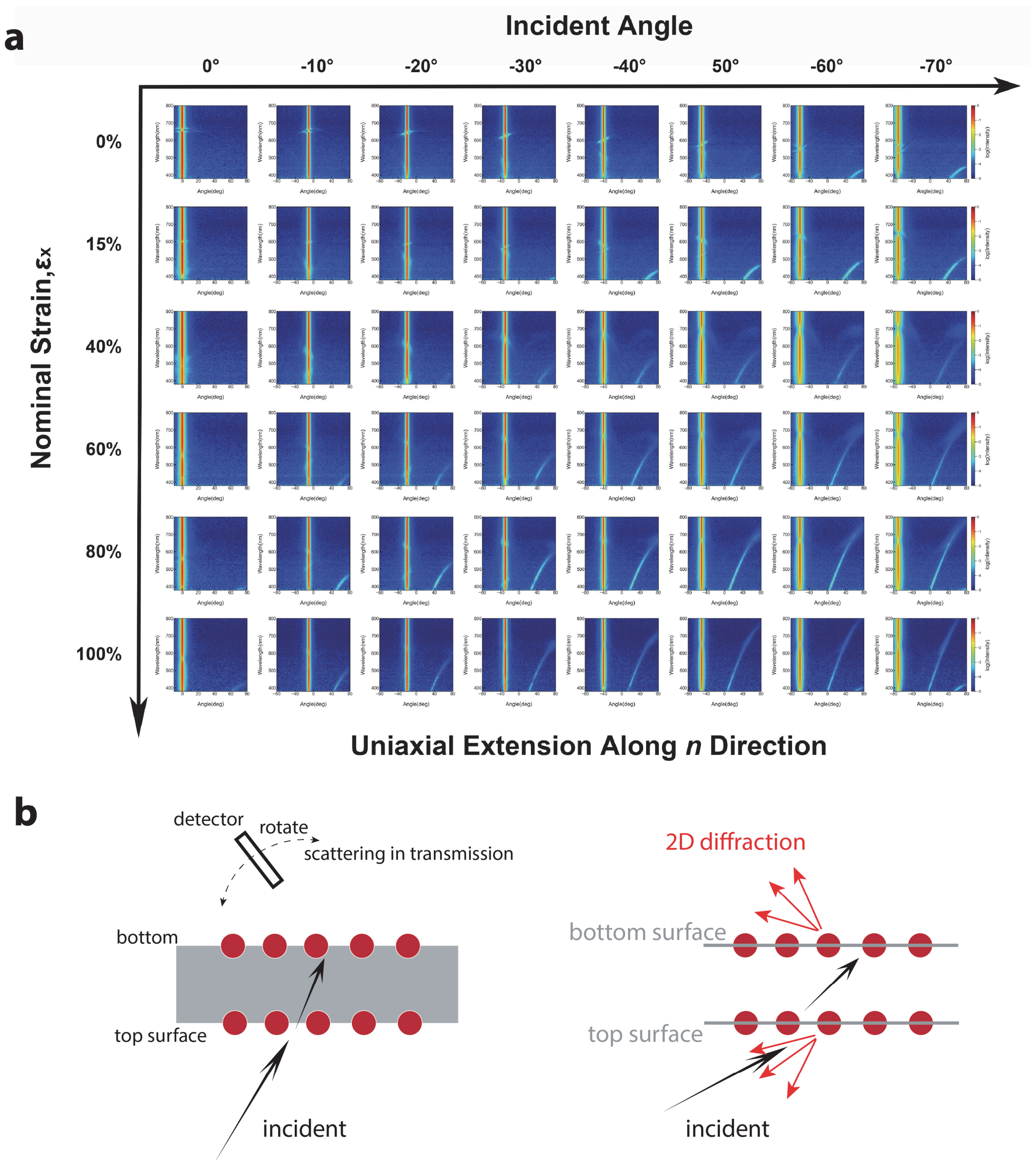

Figure S23. Angular scattering spectra in the transmission direction for *n*-stretched new red elastic opal varying in incidence angle and strain. a, Measured spectra of scattering light from the bottom surface side of the sample. **b**, the setup and the mechanism of the scattering at the bottom surface side of the sample. Left, an illustration showing the setup and the measurement geometry, right, an illustration showing that the incident light should trigger surface diffraction at both the top and bottom surfaces of the sample.

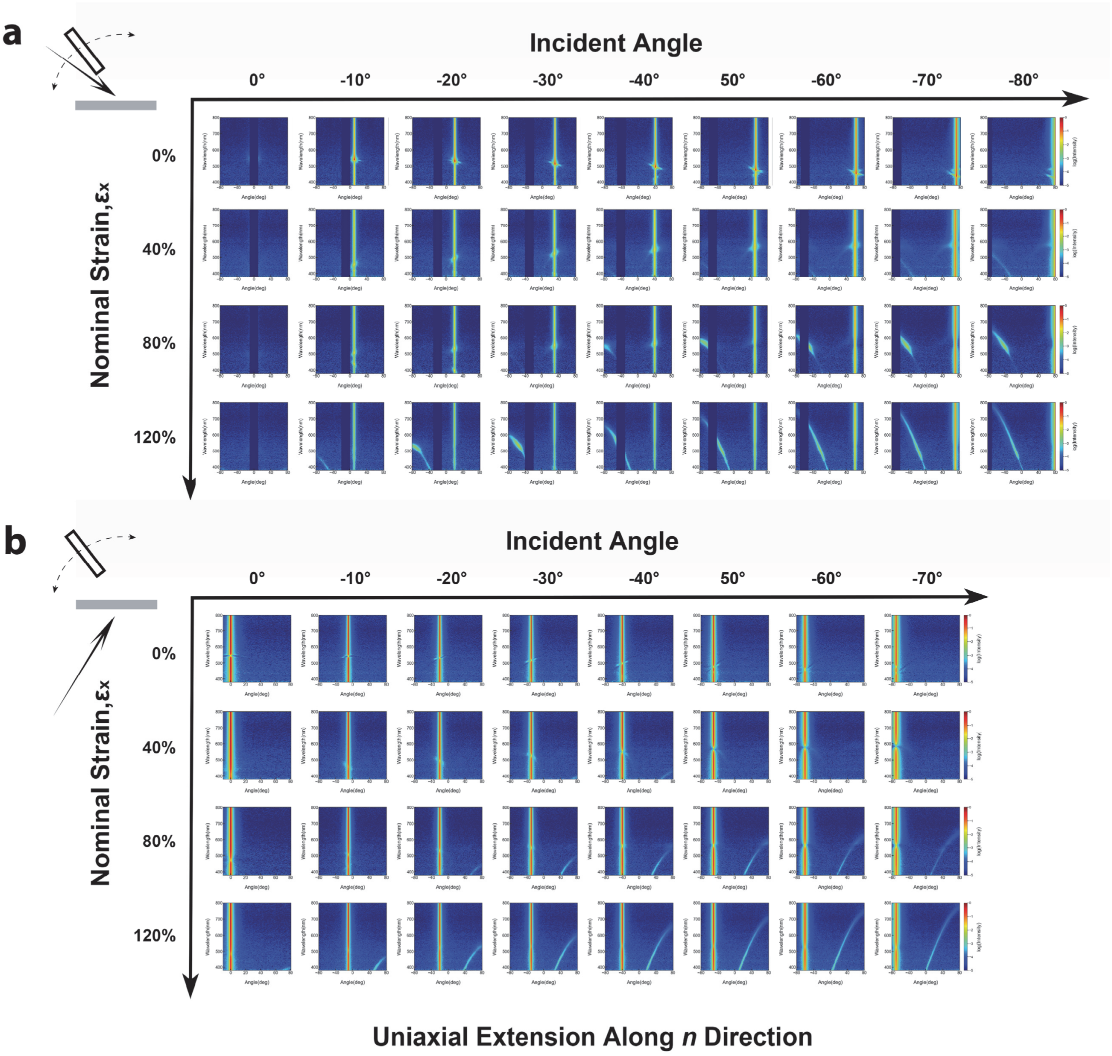

Figure S24. The angular scattering spectra of the *n*-stretched new green elastic opals in reflection and transmission. **a**, spectra at the top surface side. **b**, spectra at the bottom surface side. The peak wavelength of the new green elastic opal at normal incidence before stretching is ~ 550nm, which is similar to that of the POs used in this work.

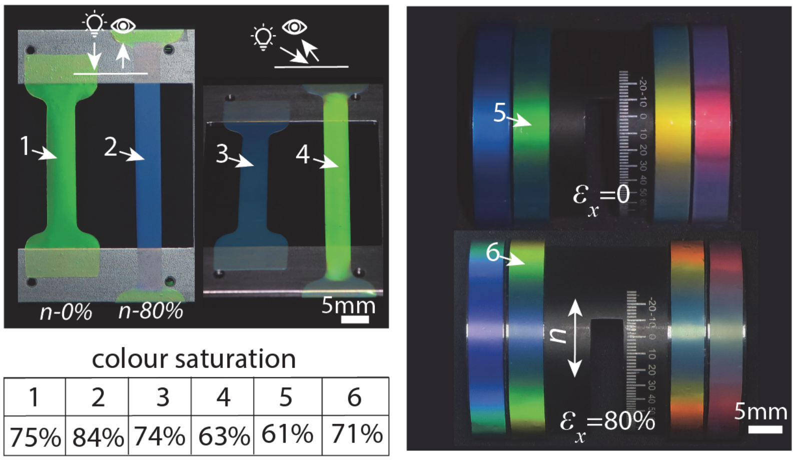

colour saturation

1	2	3	4	5	6
75%	84%	74%	63%	61%	71%

Figure S25. Colour saturation values of different colours of POs. Values of colour saturation are obtained in HSB mode in photoshop. 1 and 2 indicate the reflection colours at normal incidence before and after stretching, respectively. 3 is the colour of PO before stretching in retro-reflection, and 4 is the

bright retro-reflection colour after stretching. 5 is also the normal reflection colour before stretching, and 6 is the retro-reflection colour after stretching.

Supplementary Discussion 1. Mechanical model for stretching-induced lattice transformations

For n-stretching, $\varepsilon_z = \frac{1}{(1+\varepsilon_x)} - 1$, based on two boundary conditions: i. $V_{ABCD} = V_{A\prime B\prime C\prime D\prime}$, representing the unit cell volume pre- and post-stretching; ii. $\varepsilon_y = 0$, given that the rows of spheres aligned in the width direction resist with decrease. Position of the spheres are deduced using an additional boundary condition: $\frac{A\prime B\prime}{AB} = \frac{A\prime C\prime}{AC} = \frac{A\prime D\prime}{AD} = 1 + \varepsilon_m$, signifying equal distance between the top and the base spheres. It's unknown whether the spheres keep a constant distance (ε_m=0), or if this distance varies (ε_m≠0) due to elasticity. Models typically assuming metal plasticity theories or solid-solid transitions presuppose that spheres constantly touch, leading to ε_m=0. However, when combining the above boundary conditions, we obtain $(1+\varepsilon_x)\frac{\sqrt{3}}{2} = (\frac{3}{4}(1+\varepsilon_m)^2 - \frac{2}{3}(1+\varepsilon_z)^2)^{1/2} + ((1+\varepsilon_m)^2 - \frac{2}{3}(1+\varepsilon_z)^2)^{1/2}$, and a non-zero ε_m that varies with ε_x (Fig. S8), indicating dynamic interparticle distances. In a plasticity model using ε_m =0, the strains conform to $(1+\varepsilon_x)\frac{\sqrt{3}}{2} = (\frac{3}{4} - \frac{2}{3}(1+\varepsilon_z)^2)^{1/2} + (1 - \frac{2}{3}(1+\varepsilon_z)^2)^{1/2}$.

For p-stretching, we use two boundary conditions: i. preservation of volume; ii. equivalency of (111) and $(\bar{1}11)$ during p-stretching. We obtain $(1+\varepsilon_z) = \frac{(1+\varepsilon_n)\sqrt{3(1+\varepsilon_y)^2 - (1+\varepsilon_n)^2}}{\sqrt{2}(1+\varepsilon_y)}$ and $(1+\varepsilon_x) = \frac{\sqrt{2}}{(1+\varepsilon_n)\sqrt{(1+\varepsilon_y)^2 - (1+\varepsilon_n)^2}}$, where ε_n is the fractional separation change between spheres A and D. Strains can be computed by assuming ε_n=0. Although realistically, $\varepsilon_n$likely ranges from ~0-10%, its omission has minimal impact on strain curves.

Supplementary Discussion 2. **Bragg reflection coupled diffraction model**

The light path is illustrated in Figure 4c and Figure S12.

The light-structure interactions lie in two aspects. First, incident light is diffracted by the (111) gratings, resulting in its zero-order diffraction in the specular reflection direction (PR) and first-order diffraction over a range of angles depending on the wavelength (PD). The zero-order diffraction is the Bragg reflection of the (111) planes, originating from its inter-layer periodicity. Second, light entering the material triggers the Bragg reflection of the tilting planes, which is then sent back to the material's internal surface. During this interim, the Bragg-reflected light is also diffracted by the (111) gratings, resulting in zero-order diffraction in the original traveling direction and a diverted first-order diffraction.

Diffraction of (111) gratings is decided by:

$$n_1(\sin\varphi_1 + \sin\theta_d) = m\frac{\lambda}{q}$$

where n_1 is the refractive index of air, $\varphi_1$is the incident angle, θ_d is the diffraction angle, m is diffraction order, λ is wavelength, and q is the period of the sphere lines.

Refraction of incident light is decided by the Snell's law:

$\varphi_2 = \sin^{-1}\frac{n_1}{n_2}\sin\varphi_1$

Bragg reflection wavelength of the left tiling plane is:

$\lambda = 2n_2 d_{hkl}\cos(\theta - \varphi_2)$

For right-tilting plane is:

$\lambda = 2n_2 d_{hkl}\cos(\theta + \varphi_2)$

with θ being the tilt angle.

The Bragg-reflected light from tilted planes reflects back to the sample surfaces at angles:

$\psi_1 = 2\theta - \varphi_2$ for left tiling, and

$\psi_1 = 2\theta + \varphi_2$ for right tilting.

The critical angle for refraction determined by Snell's law is:

$\psi_c = \sin^{-1}\frac{n_1}{n_2}$

The ψ_1 for right tilting plane is always larger than ψ_c, thus no refraction occurs. For left titling planes, when $\psi_1 < \psi_c$, angle of refracted light is:

$\psi_2 = \sin^{-1}\frac{n_1}{n_2}\sin\psi_1$

Using the calculated Bragg reflection wavelength of the tilting planes in the diffraction equation for 1st-order diffraction:

$\sin\theta_d = \frac{n_2}{n_1}[2\sin\theta\cos(\theta - \varphi_2) - \sin\varphi_2] = \frac{n_2}{n_1}\sin(2\theta - \varphi_2)$

From this, $\theta_d = \psi_2$, proving that the retro-Bragg reflection of tilted planes coincides with the 1st-order diffraction of incident light. Regardless of whether refraction takes place, the 1st-order diffraction of Bragg reflection from a tilted plane always exits the surface. Similarly, its exit angle $\psi_e = -\varphi_1$, proving that it is always along the specular reflection direction of the incident light. Angular reflection spectra for *n*-stretched samples were derived by integrating this optical model with strains, lattice distances, and tilting angles computed via the lattice deformation model.

Supplementary Reference

1. White, R. P. & Lipson, J. E. G. Why Volume and Dynamics Decouple in Nanocomposite Matrices: Space that Cannot Be Accessed is Not Free. *Phys Rev Lett* **131**, 018101 (2023).
2. Gilormini, P., Toulemonde, P. A., Diani, J. & Gardere, A. Stress-strain Response and Volume Vhange of a Highly Filled Rubbery Composite: Experimental Measurements and Numerical Simulations. *Mechanics of Materials* **111**, 57–65 (2017).
