# Supplementary material for: Strain to shine: stretching-induced three-dimensional symmetries in nanoparticle-assembled photonic crystals
Source: Nat Commun. 2024 Jun 18;15:5215. doi: 10.1038/s41467-024-49535-z (PMC11189559; doi:10.1038/s41467-024-49535-z)
Supplement: Supplementary file 3 — Description of Additional Supplementary Files [file 41467_2024_49535_MOESM3_ESM.pdf]

Description of Additional Supplementary Files

File Name: Supplementary Movie 1

Description: Stretching induced retro-reflection colours.

File Name: Supplementary Movie 2

Description: In-situ scattering colour imaging in *n*-stretching. Incident along *n*-direction at 70˚, the stretching strain is indicated at the corner.

File Name: Supplementary Movie 3

Description: Reducing surface diffraction with oil. The experiment uses a new elastic opal stretched by ~80% in *n*-direction, its original peak reflection wavelength is ~ 610nm. One side is bonded to a VHB substrate and positioned on a cylinder so to we can view the retro-reflection colours from the top. The sample show retro-reflection colours from both surface diffraction and the Bragg reflection of tilted planes. The surface diffraction colour is much more pronounced compared to POs since the polyacrylate matrix marginally weakens the bond between the sphere and the matrix, leading to a significant increase in the 'pop-up' height of spheres. This amplifies the surface diffraction effect, as indicated by its blue hue. When its surface is submerged in dimethicone oil, which has a refractive index nearing 1.4, the blue surface diffraction colour becomes significantly weak.
